# Proving of Terebinthina

**Published:** 1897-06

**Authors:** E. V. Ross

**Affiliations:** Rochester, N. Y.


### PROVING OF TEREBINTHINA.

E. V. Ross, M. D., Rochester, N. Y.

A gentleman of very dark complexion informed me that whenever he came in contact with turpentine he experienced the following effects:

1. Frequent desire to urinate, but must wait some time before urine will pass.

2. Sometimes when the urine is about half voided it suddenly stops, and in a few moments starts again, and is then accompanied by a burning pain from glans penis back to perineum.

3. Burning in urethra from before backwards on urinating only.

4. Occasionally urine can be passed only during stool. (Comp. *Aloes*, *Alumina*.)

More abundant during stool.* *Amon-m.*, *Ox-ac.*—Lippe.

Can only pass urine when standing. *Sass.*

Can only pass urine when in a sitting posture. *Zinc.*

Then quite a number of remedies having an interrupted flow.

---

* Must have stool every time he passes urine. Muriatic-acid.
When urinating stool escapes. Squill.
Urine flows only during stool. Aloes, Alum.
Urine flows more abundantly during stool. Amm-mur., Oxalic-acid.—[Ed.]

